# A Scoping Review on Biopsychosocial Predictors of Mental Health among Older Adults

**DOI:** 10.3390/ijerph191710909

**Published:** 2022-09-01

**Authors:** Nia Murniati, Badra Al Aufa, Dian Kusuma, Sudijanto Kamso

**Affiliations:** 1 Doctoral Program in Public Health, Faculty of Public Health, Universitas Indonesia, Depok 16424, Indonesia; 2 Applied Health Science Department, Vocational Education Program, Universitas Indonesia, Depok 16424, Indonesia; 3 Centre for Health Economics & Policy Innovation, Imperial College Business School, London, SW7 2AZ, UK; 4 Department of Biostatistics, Faculty of Public Health, Universitas Indonesia, Depok 16424, Indonesia

**Keywords:** aged, biological factors, sociological factors, psychological factors, mental health

## Abstract

This review aims to map the available evidence on biopsychosocial predictors of elderly mental health. The articles were independently screened in three selected databases, namely Pubmed, Proquest and Google Scholar. The stages consist of identifying the research questions, seeking and selecting relevant evidence, mapping data, and concluding and reporting results. The PRISMA flowchart was used to show the PEOS evidence search flow. A total of 23,722 articles were obtained from all databases during the initial search, where 458 titles fulfilled the eligibility criteria at the title screening stage. Furthermore, 383 articles passed through abstract screening, where 75 met the inclusion criteria and were included for full-text screening. Based on the full-text screening stage, 28 articles were excluded and the remaining 47 articles that matched the search process were included for data extraction. This review creates biopsychosocial variables related to the mental health of the elderly. The biological factors consist of age, biomarkers, female, health conditions, chronic diseases, and physical function. Variables related to psychological factors are affect, personality traits, and subjective well-being. Meanwhile, social factors include smoking, sleep quality, physical activity, daily living, social support, marital status, loneliness, religiosity, spirituality, and early life conditions.

## 1. Introduction

The proportion of populations aged 60 years and above has increased globally due to the improvement in life expectancy in developing and developed countries [1,2]. It has been predicted that by 2050, the world’s population aged 60 years and above would be 1.5 billion, which corresponds to 16% of the present value [3]. Older people contribute to society in many ways within their families, local communities, or the community more broadly [4,5]. However, declination of physical and mental capacities that might lead to the poor health condition of the elderly population can negatively affect the health, economics, and social aspects of life [4,6].

Mental and neurological disorders (excluding headaches) affect more than 20% of adults 60 and older, and 6.6% of all disabilities (disability-adjusted life years—DALYs) can be attributed to these disorders [7]. This age group is most commonly affected by dementia, which affects approximately 5% to 7% of the world’s elderly population. Around a quarter of all deaths from self-harm are caused by anxiety disorders, which affect 3.8% of the elderly population, and substance use problems, which are often neglected or misdiagnosed, influence approximately 1% [7].

The biopsychosocial model has been prominently used in examining determinants of mental health [8]. A variety of factors affecting mental health and illness, such as genetics, infections, physical traumas, nutrition, hormones, and environmental toxins, are all considered in this model [9]. For example, psychological experts might look for signs of poor self-control, anxiety, or a tendency toward negative thinking as causes of a health issue. Layoffs are a wide range of stressful life events that can have varying effects on one’s mental health depending on the individual and the surrounding social context [10]. Patients and clinicians’ dyadic relationships, as well as multidisciplinary approaches to health care, benefit from the model’s insights into biological and psychosocial aspects of illness [11].

The variations in individual characteristics between the young and old age groups lead to a difference in the biopsychosocial predictor scope [12,13]. The investigation of the biopsychosocial model to categorize mental health risk factors in older adults is often without clear boundaries. This makes the mapping and synthesizing of a collection of literature essential to identify trends and gaps in the scientific reports. It can also assist in the development of a biopsychosocial model as a tool to identify determinants of mental health in older adults. Therefore, this research aims to map the available evidence on biopsychosocial predictors of mental health in older adults that have been measured in previously published articles.

## 2. Materials and Methods

### 2.1. Design

Scoping review of the literature has been carried out by identifying the types of available evidence and key factors related to the biopsychosocial mental health of the elderly [14]. A total of 5 steps suggested by Tricco et al. were adopted in this research, (1) identify the research question, (2) identify relevant previous research, (3) select using an iterative team approach, (4) chart the data by summarizing quantitative data and qualitative thematic analysis, and (5) collate, summarize, and report the results [15]. However, recommended consultation with stakeholders was not implemented since it is considered an optional component of scoping reviews [15,16]. A review protocol was also not involved in the registry and the quality of the article or any bias risk was not calculated since this research was a scoping review [16,17].

### 2.2. Identifying the Research Question

This scoping review aims to answer the following question “Which biopsychosocial aspects as predictors of mental health in the older adult were measured in previously published research?”. To develop the focus of the review and search strategy, the Population, Exposure, Outcome, and Study design (PEOS) framework was used as shown in Table 1 [18]. Furthermore, PEOS can assist in developing suitable search terms to describe the problem and define inclusion as well as the exclusion criteria [19].

### 2.3. Search Strategy

Relevant published articles from 7 September 2021, to 30 May 2022 were searched using a computer, in various databases such as Pubmed, Proquest and Google Scholar. The date of the article was from 2017 to 2022 and in the English language and only quantitative research using internationally recognized criteria for assessing body composition was included. However, it excluded articles on evidence on biopsychosocial factors of mental health among adolescent or adult people, non-English language, systematic reviews, and meta-analyses. To find relevant published articles, a full keyword search was performed with Boolean AND/OR, while Mesh Term and subheading were used in PubMed. The Preferred Reporting Items for Systematic Reviews and Meta-Analysis (PRISMA) flowchart was used to fully and transparently describe the process of identifying articles [17]. The keywords used in the search for articles were *“Biological Factors” OR “psychological factors” OR “Social Factors” AND “Mental Health” AND “older adult”*.

This research includes articles on evidence of biopsychosocial factors associated with mental health in the elderly and only English articles published between 2017 and 2022. The inclusion criteria only reviewed original articles that were quantitatively designed without geographical limitations. The exclusion criteria were review articles and books, while those included in the search were imported to Mendeley Desktop with duplicate articles removed. The abstract and full-text screening process used inclusion and exclusion criteria. The differences after abstract screening were resolved through discussion to build consensus and the adapted PRISMA flowchart was used to document the selection process.

### 2.4. Charting Data

The data were extracted by using Microsoft Word and the elements of the extraction involved title, author, date, objective, design, setting, sampling, variables, data analysis, and outcomes reported.

### 2.5. Collating and Summarizing the Results

After the data extraction, a thematic content analysis was conducted and the results were summarized and coded manually. The articles were coded into the following themes, namely biological, psychological, and social factors as presented in Table 2.

## 3. Results

### 3.1. Literature Search

**Figure 1:**
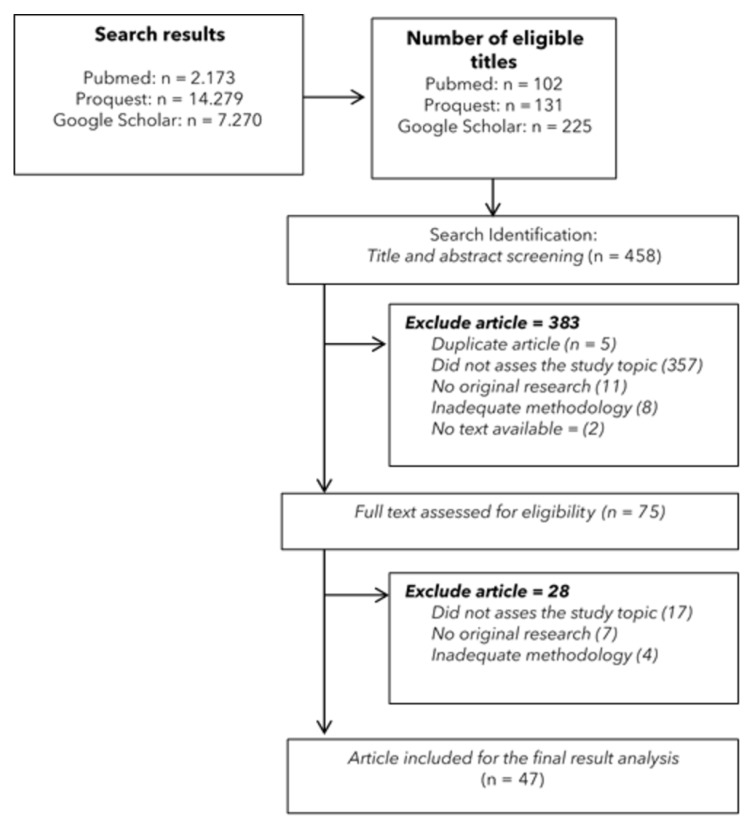
A flow diagram of research selection.

During the initial search, 23,722 articles were retrieved from all databases, with 458 titles meeting the screening criteria. In Mendeley Desktop, which was used to gather eligible titles from databases, all five titles identified as duplicates were removed. After the abstract screening, 383 articles were selected, of which 75 met the inclusion criteria and were moved on to the full-text screening stage. Full-text screening revealed that 28 articles were ruled out and the remaining 47 were included for data extraction as shown in Figure 1. Table S1 shows a summary of characteristics of the identified studies.

### 3.2. Research Characteristics

It has been observed that research related to biopsychosocial factors of mental health in the elderly increased annually from 4 to 11 articles between 2017 and 2021. The peak occurred in 2020 when there were 15 published articles related to the topic. Based on the location, most research was conducted in Asia and Europe with 18 and 15 results, while the least was in Australia and Africa with 3 and 2 results, respectively (Table 3).

## 4. Discussion

The biopsychosocial model was created as an effort to complement the existing biomedical one [20]. This is because the biomedical model has limitations and fails to consider the psychological and social factors that contribute to disease as well as health [21]. The traditional biomedical view showed that biological indices were the main criteria for defining disease, while paradoxically finding some people that felt sick when they did not have the “disease” or vice versa [9]. The biopsychosocial model can explain why some individuals experience a state of “illness” that others perceive as simply a “problem of living”, such as emotional reaction to living circumstances or somatic symptoms. The perspective of the decision on a person with the “problem of living” or “illness” is related to whether people accept the role of being sick and trying to get into the health care system. Some individuals also deny the reality of unwanted illness by ignoring symptoms that are indicative of serious organic processes [9].

Health, disease, and healthcare have all been defined by the biopsychosocial paradigm since the 1970s [8]. The model recommends that health facilities consider these three aspects by integrating multidisciplinary science in providing effective health services [22,23]. Furthermore, Engel suggested its use according to the scientific content and clinical applications. The empirical methodology has proven that the biopsychosocial model can show causal relationships with health outcomes [8].

The models are commonly used in chronic conditions, which are assumed to be psychophysiological behavioral patterns that are not categorized into biological, psychological, or social factors [24]. There are many factors that influence health and disease, but the biopsychosocial model proposes a more complex way of understanding how these factors interact to influence health [25].

### 4.1. Biological Factors

Biological factors are the same materials and processes as the genetic characteristics of parents, which include the function and structure of a person’s physiology [26]. The body includes very complex physical systems, where every organ, bone, and nerve are made up of tissues composed of various cells, molecules, and atoms [27]. The healthy functioning of this system depends on the mutual operation and interaction of each component [25].

One of the biological factors that influence mental health is age. It has been discovered that older age is a risk factor for cognitive impairment, mental health, and life quality [28,29,30]. An aging body experiences involutional changes in various systems and organs, which leads to a decrease in overall efficiency, the coexistence of diseases, and changes in symptomatology [31]. With age, memory and cognitive functions decline, and there is a greater risk of depression and stupefaction [31].

Research in Spain, Canada, and England has shown that elderly women experience more psychological distress than men [32,33,34]. Psychological distress has a considerable impact on the social functioning of parents, and gender is a relevant predictor of depression [35]. Moreover, depression accounts for 10% of the total burden of non-fatal disease worldwide, which falls disproportionately on women [36]. Recent estimates of the point prevalence of depression in women are 5.9%, while men are 3.8% [37]. In Lithuania, it has been confirmed that women show a higher risk of developing anxiety disorders and/or depression compared to men [38]. Another important discovery has stated that there are different biological patterns in the gender of individuals experiencing depression, where women showed higher and significantly correlated levels of inflammatory, neurotrophic, and serotonergic markers than men [39]. This confirmed the possibility of different biological patterns for men and women with depressive disorders.

In Ireland, it has been stated that the physical health condition of the elderly is significantly associated with anxiety and depression [40]. Other research has shown that health conditions with chronic disease are good predictors of perceived life expectancy, which directly affects life quality [30,41]. Furthermore, there is a list of major disease conditions as one of the components of successful aging in the elderly [42].

Physical function is the ability to perform motor tasks that involve complex integration of several physiological systems such as the neuromotor, musculoskeletal, and cardiorespiratory systems [43,44]. When the elderly experience a decline in physical function, they face difficulty in engaging in activities of daily living and try to avoid or limit this activity. The decline in physical function can occur gradually, subtly, and may not be immediately visible to health care providers, families, or even individuals until the person is unable to perform activities at all [43]. Research has shown that physical function in the elderly is associated with several physical and mental disorders, life quality, and a significant effect on successful aging [30,42,43].

### 4.2. Psychological Factors

Psychology is concerned with how people act and think, including their cognition, emotions, and motivations [24,45]. Mental processes such as acquisition of new information, retention of that information, reasoning, interpretation, and resolution of problems all fall under cognition [46]. Emotions are complicated interactions of subjective feelings that affect and are influenced by external stimuli [47]. There are positive emotions, such as joy and affection, and then there are negative emotions such as fear, anxiety, and sadness [48]. Positive emotional correlation is associated with better health and a quicker recovery from illness than negative correlation in a variety of ways, including decreased risk of illness and better health overall [49]. In addition, peoples’ decisions to seek medical treatment may be influenced by their emotions [50]. Motivation is a process within an individual to initiate some activity, select its direction, and sustain it [51]. Another example is parents who quit smoking because they are motivated to protect their children’s health [25].

Research in Afghanistan has shown that negative affect or aggression is associated with mental distress [29]. Meanwhile, in Italy, it has been reported that there is a weak relationship between positive affect and subjective well-being [52]. The degree to which a person subjectively feels good emotions like joy, curiosity, and alertness is referred to as positive affect [53]. According to Fredrickson, the four basic types of positive feeling are love, joy, interest, and contentment [54]. Positive affect appears to have various effects on people’s cognition and behaviors, as well as their physical and mental health and the quality of their social interactions. It also appears to have a significant impact on their level of life satisfaction [55].

Personality traits explain that most individuals have an innate tendency to experience certain moods and emotions more often or with different intensities than others [56]. The Big Five Personality Model is also known as the Five-Factor Model and measures Openness, Conscientiousness, Extraversion, Agreeableness, and Neuroticism [57]. Each individual is in the middle of a continuum and tends to be closer to one side, but still has some aspects of the opposite side [58]. It has been discovered that personality traits with low extraversion and conscientiousness, and high neuroticism predict depressive symptoms [59]. Another personality characteristic related to psychological resilience is grit, which is defined as “perseverance for long-term goals and passion” [60]. A recent investigation in adults with LLD stated that grit was associated with reduced severity of depression, apathy, and anxiety [61].

An individual’s cognitive and affective assessment of his or her life is called subjective well-being (SWB), and it is primarily based on three factors (negative affect, positive affect and live satisfaction) [62]. A relationship has been found between the lifestyle of the elderly and SWB [52]. Research in China has proved that better SWB can reduce mental health problems in the elderly [63]. Moreover, in Spain and Costa Rica, SWB is associated with successful aging [64].

### 4.3. Sociological Factors

Humans live in a social world having relationships with other individuals such as family members, friends, or groups, where someone’s interaction with other people influences one another. Social processes provide a strong motivation that can be used as an important predictor of future health. At a fairly broad level, the social environment affects individual health by promoting certain cultural values such as being fit and healthy, or a larger social unit [25].

Smoking has been reported to affect perceived life expectancy, mental health, and subjective well-being [41,63,65]. Mentally ill people are more likely than the general public to begin smoking at a younger age, smoke more frequently, and become addicted to cigarettes [66]. Those with schizophrenia, bipolar disorder, depression and anxiety who smoke are more likely to experience severe symptoms and require higher doses of some psychotropic drugs than individuals who do not smoke [67].

Research has shown that sleep disturbances and deprivation are associated with chronic disease, decreased quality of life, and high use of health facilities [68,69,70]. The elderly generally has a higher prevalence of chronic diseases and sleep disorders. Some evidence has suggested that the biological need for sleep can decrease with age [71]. This shows the possibility that increased irregular sleep will exacerbate the disease process [72]. Other results have proved that sleep difficulties have a significant effect on anxiety [40]. The patterns are also a component of a healthy lifestyle that can foster positive feelings and lead to better subjective well-being [63].

The “activities of daily living” (ADL) refers to a group of core abilities needed for self-care on one’s own [73]. Measuring a person’s ability to carry out basic daily functions like eating, getting dressed, bathing, and toileting are all part of the ADL process [74]. Individuals who have difficulty carrying out activities of daily living are more likely to experience depression [40].

Social support refers to the comfort, attention, appreciation, or assistance available to a person from another individual or group [75]. Social support provides health benefits through cooperative norms or informal networks [76]. This is to increase interaction with other people, create, and develop social norms, environmental reciprocity, as well as social trust, which promote communication and cooperation among community members. Furthermore, social support, as measured by mutual trust in the community, shows a positive relationship with the physical and mental health of the elderly in eastern Indonesia [74].

Research in Ireland has shown that lack of involvement in the community is associated with depression and anxiety in the elderly [40]. Similarly, in South Africa, it has been discovered that elderly people who have difficulty joining the community are more likely to experience depression and lose interest [77]. In China, participation in the community affects the successful aging of the elderly [42].

One of the significant social factors that affect depression is marital status. This is because married individuals have better mental health than those who are single, widowed, separated, and divorced [78]. Being married is a protective factor for depression while being unmarried is a significant risk factor for depression in the elderly/late-life [79]. Other research has shown the positive implications of marriage by increasing subjective well-being and reducing loneliness, anger, symptoms of chronic depression, and stress [80]. In older adult, the spouse helps their spouse with physical limitations and provides the most of the assistance and care [81]. With the help of their wives, married men with functional limitations saw a decrease in their depressive symptoms, but the relationship was lost for women [82]. The unmarried elderly typically experience longer periods of loneliness and lack of social support, which makes them more prone to depression [83]. The death of a spouse significantly changes the lifestyle and psychological state, which makes the depression risk higher in the elderly [84].

A consensus has stated that religiosity involves beliefs, practices, and rituals associated with the sacred as shown by observing feelings, behaviors, and experiences [85,86]. This includes organized participation in community and private practices/rituals. It is common to view spirituality as a broad concept that can be defined by each individual and is not necessarily associated with any particular organized religion [87]. Religiosity and spirituality are common phenomena in elderly life. This is because seeking answers to the meaning of life in religiosity and spirituality provides a sense of well-being, reduces anxiety and helplessness, and increases resilience to situational problems as well as difficulties associated with aging [88,89,90]. Individual spirituality can also be expressed through values, beliefs and ritual practices for the pursuit of transcendence, self-reflection, and thoughts about existential relationships outside the objective world [91].

Spirituality is a complex multidimensional concept described as the way individuals perceive and experience life, which acquires its highest meaning and value as a subjective impact of the sacred, as well as qualities that transcend religious affiliation, such as inspiration, respect, admiration, meaning, and purpose, even in those who believe in nothing [92]. Spirituality has four characteristics, namely relationship with fellow human beings, oneself, nature, and God/the highest power [93]. Out of these characteristics, spirituality is generally divided into vertical and horizontal aspects, namely the connection with God/the highest power and the relationship with created beings [94]. It showed that spirituality has a significant positive impact on subjective well-being [52]. Other results have discovered that spiritual well-being is directly related to depression, which is significantly affected by functional ability [95]. In Portuguese, it has been stated that spirituality has a direct effect on the mental health of the elderly [96].

As an unstructured, personal, and spontaneous phenomenon, spirituality can be defined as a person’s search for a sense of belonging and connection with a higher power or purpose [97]. Transcendence is directed to a higher entity or energy or as a creator God [98]. The other component of spirituality is the relationship with created beings, namely oneself, other people, and nature/environment [93]. This creates harmonization in life that encapsulates the general understanding of relationships with other people such as caring for, taking care of, sharing, and getting along with others [94].

The long-term effect of early life conditions on mental health is shown at an older age through different mechanisms. Exposure to disease or the context of economic deprivation has been discovered to have lasting consequences on mental health [99]. Research in China has proved that childhood adversity affects cognitive function among the middle-aged population [100]. Other results have shown that individuals who experienced many adverse childhood experiences in early childhood are at risk for depression, anxiety, substance abuse habits, and adverse mental health behaviors as adults [101]. This suggests that early countermeasures for childhood adversity can lead to the effective reduction of cognitive impairment [100]. Therefore, social policies need to consider social conditions from an early age to promote mental health and healthy aging strategies [102].

As of the time of writing, the COVID-19 pandemic is still ongoing. Obviously, the COVID-19 epidemic affects mental health, even that of the elderly. During the COVID-19 pandemic, numerous older groups felt anxious and under strain. They feared being ill and dying, avoided health care institutions for fear of infection, and felt powerless to protect their loved ones. Loneliness and feelings of isolation during quarantine, restrictions on outdoor mobility and limited mastery of online applications, and unfamiliarity with the use of personal protective equipment or other methods of preventing COVID-19 transmission are sources of psychological distress for the elderly [103]. However, the pandemic brought about conditions that were significantly distinct from the norm. Therefore, we considered excluding papers pertaining to the impact of the pandemic on the mental health of the elderly, as the discourse provided could vary between pandemic and non-pandemic situations. Therefore, further reviews can be carried out to particularly find out why and how the pandemic has affected the mental health of older people. Moreover, although the role of biological, psychological, and social factors influencing health and disease is not difficult to examine, it is more complex to understand how health is affected by the interaction of these components. Therefore, further research is needed to examine the pattern of interaction between biological, psychological, and social factors to explain the direct or indirect effect on the mental health of the elderly.

## 5. Conclusions

The biopsychosocial model is suitable as a framework for thinking because mental health needs to be holistic. It is widely used in the medical field, specifically in psychiatry. Previous research has explained that a series of biopsychosocial factors that change with age can predict mental health in the elderly group. The findings from this scoping review mapped the risk of mental health from biological factors consisting of age, sex, physical function, health conditions, and chronic illness. Psychological factors consist of affect, personality traits, and subjective well-being. The risk to mental health in the elderly comes from social factors like smoking, physical activity, quality of sleep, daily activities, social support, marital status, loneliness, religion and spirituality, and conditions in early life.

## Figures and Tables

**Table 1:** PEO Framework for determining the eligibility of the scoping review question.

Population	Elderly, Older adults
Exposure	Biological, psychological and social factor
Outcomes	Mental health, mental illness
Research Design	Quantitative

**Table 2:** Code and theme extracted of literatures reviewed.

Theme	Sub Theme
Biological Factor	Age Gender-Women Health conditions and chronic illness Physical function
Psychological Factors	Affect Personality Traits Subjective well-being
Social Factor	Smoking behavior Physical activity Sleep quality Activities of daily living Social support Marital status and loneliness Religiosity and spirituality Early life conditions

**Table 3:** Research characteristics.

Characteristics	n	%
**Year**		
2017	4	8.5
2018	6	12.8
2019	8	17.0
2020	15	31.9
2021	11	23.4
2022	3	6.4
**Continent**		
Africa	3	6.4
America	5	10.6
Asia	18	38.3
Australia	2	4.3
Europe	15	31.9
Inter-continent	4	8.5

## Data Availability

Not applicable.

## Supplementary Materials

The following supporting information can be downloaded at: https://www.mdpi.com/article/10.3390/ijerph191710909/s1, Table S1. Summary of Characteristics of Identified Studies.

## References

[1] World Health Organization (2022). Ageing and Health. https://www.who.int/news-room/fact-sheets/detail/ageing-and-health#:~:text=Atthistimetheshare,2050toreach426million.

[2] World Health Organizaton (2020). WHO Methods and Data Sources for Life Tables 1990–2019.

[3] United Nations Department of Economic and Social Affairs (2020). World Population Ageing 2019 [Internet].

[4] World Health Organization (2015). World Report on Ageing and Health [Internet].

[5] Cristea M., Noja G.G., Stefea P., Sala A.L. (2020). The Impact of Population Aging and Public Health Support on EU Labor Markets. Int. J. Environ. Res. Public Health.

[6] Maresova P., Javanmardi E., Barakovic S., Barakovic Husic J., Tomsone S., Krejcar O., Kuca K. (2019). Consequences of chronic diseases and other limitations associated with old age–a scoping review. BMC Public Health.

[7] World Health Organizaton (2017). Key Facts: Mental Health of Older Adults. https://www.who.int/news-room/fact-sheets/detail/mental-health-of-older-adults.

[8] Bolton D., Gillett G. (2019). The Biopsychosocial Model of Health and Disease: New Philosophical and Scientific Developments.

[9] Engel G.L. (1977). The Need for a New Medical Model: A Challenge for Biomedicine. Science.

[10] Gopalkrishnan N. (2018). Cultural Diversity and Mental Health: Considerations for Policy and Practice. Front. Public Health.

[11] Kusnanto H., Agustian D., Hilmanto D. (2018). Biopsychosocial model of illnesses in primary care: A hermeneutic literature review. J. Fam. Med. Prim. Care.

[12] Beerbower E., Winters D., Kondrat D. (2018). Bio-psycho-social-spiritual needs of adolescents and young adults with life-threatening illnesses: Implications for social work practice. Soc. Work. Health Care.

[13] Rook K.S., Charles S.T., Heckhausen J. (2007). Aging and Health. Foundations of Health Psychology.

[14] Munn Z., Peters M.D.J., Stern C., Tufanaru C., McArthur A., Aromataris E. (2018). Systematic review or scoping review? Guidance for authors when choosing between a systematic or scoping review approach. BMC Med. Res. Methodol..

[15] Ricco A.C., Lillie E., Zarin W., O’Brien K., Colquhoun H., Kastner M., Levac D., Ng C., Sharpe J.P., Wilson K. (2016). A scoping review on the conduct and reporting of scoping reviews. BMC Med. Res. Methodol..

[16] Arksey H., O’Malley L. (2005). Scoping studies: Towards a methodological framework. Int. J. Soc. Res. Methodol..

[17] Peters M.D.J., Godfrey C.M., Khalil H., McInerney P., Parker D., Soares C.B. (2015). Guidance for conducting systematic scoping reviews. Int. J. Evid. Based Healthc..

[18] Danquah F., Ansu-Mensah M., Bawontuo V., Yeboah M., Kuupiel D. (2020). Prevalence, incidence, and trends of childhood overweight/obesity in Sub- Saharan Africa: A systematic scoping review. Arch. Public Health.

[19] Bettany-Saltikov J. (2010). Learning how to undertake a systematic review: Part 2. Nurs. Stand..

[20] Farre A., Rapley T. (2017). The New Old (and Old New) Medical Model: Four Decades Navigating the Biomedical and Psychosocial Understandings of Health and Illness. Healthcare.

[21] Borrell-Carrió F., Suchman A.L., Epstein R.M. (2004). The Biopsychosocial Model 25 Years Later: Principles, Practice, and Scientific Inquiry. Ann. Fam. Med..

[22] Kamper S.J., Apeldoorn A.T., Chiarotto A., Smeets R., Ostelo R., Guzman J., van Tulder M. (2015). Multidisciplinary biopsychosocial rehabilitation for chronic low back pain: Cochrane systematic review and meta-analysis. BMJ.

[23] Maltzman S. (2016). A multidisciplinary, biopsychosocial approach to treatment: Implications for research and practice. The Oxford Handbook of Treatment Processes and Outcomes in Psychology: A Multidisciplinary, Biopsychosocial Approach.

[24] Gatchel R.J., Peng Y.B., Peters M., Fuchs P., Turk D.C. (2007). The biopsychosocial approach to chronic pain: Scientific advances and future directions. Psychol. Bull..

[25] Sarafino E.P., Smith T.W. (2017). Health Psychology: Biopsychosocial Interactions.

[26] Hernandez L.M., Blazer D.G., Institute of Medicine (2006). Genes, Behavior, and the Social Environment: Moving Beyond the Nature/Nurture Debate.

[27] Zhang K. (2020). The Significance of Physiological Spaces in the Body and Its Medical Implications. Research.

[28] Xu W., Hu X., Zhang X., Ling C., Wang C., Gao L. (2021). Cognitive Impairment and Related Factors Among Middle-Aged and Elderly Patients with Type 2 Diabetes from a Bio-Psycho-Social Perspective. Diabetes Metab. Syndr. Obes. Targets Ther..

[29] Sancilio A., Eggerman M., Panter-Brick C. (2017). Biocultural research in global mental health: Mapping idioms of distress onto blood pressure in a population survey. Am. J. Hum. Biol..

[30] Lima S., Teixeira L., Esteves R., Ribeiro F., Pereira F., Teixeira A., Magalhães C. (2020). Spirituality and quality of life in older adults: A path analysis model. BMC Geriatr..

[31] Dziechciaż M., Filip R. (2014). Biological psychological and social determinants of old age: Bio-psycho-social aspects of human aging. Ann. Agric. Environ. Med..

[32] Matud M.P., García M.C. (2019). Psychological Distress and Social Functioning in Elderly Spanish People: A Gender Analysis. Int. J. Environ. Res. Public Health.

[33] Drapeau A., Beaulieu-Prévost D., Marchand A., Boyer R., Préville M., Kairouz S. (2010). A life-course and time perspective on the construct validity of psychological distress in women and men. Measurement invariance of the K6 across gender. BMC Med. Res. Methodol..

[34] Steptoe A., Leigh E.S., Kumari M. (2011). Positive affect and distressed affect over the day in older people. Psychol. Aging.

[35] Viertiö S., Kiviruusu O., Piirtola M., Kaprio J., Korhonen T., Marttunen M., Suvisaari J. (2021). Factors contributing to psychological distress in the working population, with a special reference to gender difference. BMC Public Health.

[36] Forber-Pratt A.J., Lyew D.A., Mueller C., Samples L.B. (2017). Disability identity development: A systematic review of the literature. Rehabil. Psychol..

[37] Ferrari A.J., Charlson F., Norman R.E., Patten S., Freedman G.D., Murray C.J., Vos T., Whiteford H. (2013). Burden of Depressive Disorders by Country, Sex, Age, and Year: Findings from the Global Burden of Disease Study 2010. PLOS Med..

[38] Serpytis P., Navickas P., Lukaviciute L., Navickas A., Aranauskas R., Serpytis R., Samalavicius R. (2018). Original Article Gender-Based Differences in Anxiety and Depression Following Acute Myocardial Infarction. Arq. Bras. Cardiol..

[39] Labaka A., Goñi-Balentziaga O., Lebeña A., Pérez-Tejada J. (2018). Biological Sex Differences in Depression: A Systematic Review. Biol. Res. Nurs..

[40] Bond L., Carroll R., Mulryan N., O’Dwyer M., O’Connell J., Monaghan R., Sheerin F., McCallion P., McCarron M. (2020). Biopsychosocial factors associated with depression and anxiety in older adults with intellectual disability: Results of the wave 3 Intellectual Disability Supplement to The Irish Longitudinal Study on Ageing. J. Intellect. Disabil. Res..

[41] Kobayashi L.C., Beeken R.J., Meisel S.F. (2017). Biopsychosocial predictors of perceived life expectancy in a national sample of older men and women. PLoS ONE.

[42] Chen X.X., Su D., Chen X.X., Chen Y. (2021). What intensity of exercise is most suitable for the elderly in China? A propensity score matching analysis. BMC Public Health.

[43] Garber C.E., Greaney M.L., Riebe D., Nigg C.R., Burbank P.A., Clark P.G. (2010). Physical and mental health-related correlates of physical function in community dwelling older adults: A cross sectional study. BMC Geriatr..

[44] Jones S., Schultz M.G., Tillin T., Park C., Williams S., Chaturvedi N., Hughes A.D. (2021). Sex differences in the contribution of different physiological systems to physical function in older adults. GeroScience.

[45] Dai D.Y., Stenberg R.J. (2004). Motivation, Emotion, and Cognition: Integrative Perspectives on Intellectual Functioning and Development.

[46] Dhakal A., Bobrin B.D. (2022). Cognitive Deficits.

[47] Tyng C.M., Amin H.U., Saad M.N.M., Malik A.S. (2017). The Influences of Emotion on Learning and Memory. Front. Psychol..

[48] Fredrickson B.L. (2001). The role of positive emotions in positive psychology. The broaden-and-build theory of positive emotions. Am. Psychol..

[49] Tugade M.M., Fredrickson B.L. (2004). Resilient Individuals Use Positive Emotions to Bounce Back From Negative Emotional Experiences. J. Personal. Soc. Psychol..

[50] Kozlowski D., Hutchinson M., Hurley J., Rowley J., Sutherland J. (2017). The role of emotion in clinical decision making: An integrative literature review. BMC Med. Educ..

[51] Cook D.A., Artino A. (2016). Motivation to learn: An overview of contemporary theories. Med. Educ..

[52] Villani D., Sorgente A., Iannello P., Antonietti A. (2019). The Role of Spirituality and Religiosity in Subjective Well-Being of Individuals With Different Religious Status. Front. Psychol..

[53] Miller D.N., Goldstein S., Naglieri J.A. (2011). Positive Affect BT-Encyclopedia of Child Behavior and Development.

[54] Fredrickson B.L. (1998). What Good Are Positive Emotions?. Rev. Gen. Psychol..

[55] Lyubomirsky S., King L., Diener E. (2005). The benefits of frequent positive affect: Does happiness lead to success?. Psychol. Bull..

[56] Robbins S.P. (2003). Organizational Behavior.

[57] Widiger T.A., Crego C. (2019). The Five Factor Model of personality structure: An update. World Psychiatry.

[58] John O.P., Srivastava S. (1999). The Big Five Trait taxonomy: History, measurement, and theoretical perspectives. Handbook of Personality: Theory and Research.

[59] Hakulinen C., Elovainio M., Pulkki-Råback L., Virtanen M., Kivimaki M., Jokela M. (2015). Personality and Depressive Symptoms: Individual Participant Meta-Analysis Of 10 Cohort Studies. Depression Anxiety.

[60] Datu J.A.D. (2021). Beyond Passion and Perseverance: Review and Future Research Initiatives on the Science of Grit. Front. Psychol..

[61] Laird K.T., Krause-Sorio B., Funes C., Lavretsky H. (2019). Psychobiological factors of resilience and depression in late life. Transl. Psychiatry.

[62] Griffin P., Ward P. (2016). Happiness and Subjective Well-Being. Encycl Ment. Health.

[63] Zhang L., Bi X., Ding Z. (2021). Health lifestyles and Chinese oldest-old’s subjective well-being—evidence from a latent class analysis. BMC Geriatr..

[64] Blanco-Molina M., Pinazo-Hernandis S., Tomás J.M. (2019). Subjective well-being key elements of Successful Aging: A study with Lifelong Learners older adults from Costa Rica and Spain. Arch. Gerontol. Geriatr..

[65] Smith T.C., Smith B., Smith T.C., Smith B. (2016). Consistency in Physical Activity and Increase in Mental Health in Elderly over a Decade: Are We Achieving Better Population Health?. AIMS Med. Sci..

[66] Fluharty M., Taylor A.E., Grabski M., Munafo M. (2016). The Association of Cigarette Smoking with Depression and Anxiety: A Systematic Review. Nicotine Tob. Res..

[67] Brose L.S., Brown J., Robson D., McNeill A. (2020). Mental health, smoking, harm reduction and quit attempts-A population survey in England. BMC Public Health.

[68] Gangwisch J.E., Heymsfield S.B., Boden-Albala B., Buijs R.M., Kreier F., Pickering T.G., Malaspina D. (2007). Sleep duration as a risk factor for diabetes incidence in a large U.S. sample. Sleep.

[69] Roth T., Ancoli-Israel S. (1999). Daytime consequences and correlates of insomnia in the United States: Results of the 1991 National Sleep Foundation Survey. II. Sleep.

[70] Kapur V.K., Redline S., Nieto F.J., Young T.B., Newman A.B., Henderson J.A. (2002). The relationship between chronically disrupted sleep and healthcare use. Sleep.

[71] Edwards B., O’Driscoll D., Ali A., Jordan A., Trinder J., Malhotra A. (2010). Aging and Sleep: Physiology and Pathophysiology. Semin. Respir. Crit. Care Med..

[72] Full K.M., Malhotra A., Crist K., Moran K., Kerr J. (2018). Assessing psychometric properties of the PROMIS Sleep Disturbance Scale in older adults in independent-living and continuing care retirement communities. Sleep Health.

[73] Edemekong P.F., Bomgaars D.L., Sukumaran S., Schoo C. (2022). Activities of Daily Living.

[74] Cao J., Rammohan A. (2016). Social capital and healthy ageing in Indonesia. BMC Public Health.

[75] Uchino B.N. (2004). Social Support and Physical Health: Understanding the Health Consequences of Relationships.

[76] Fukuyama F. (2000). Social Capital and Civil Society (April 2000). IMF Working Paper No. 00/74. https://ssrn.com/abstract=879582.

[77] Hao G., Bishwajit G., Tang S., Nie C., Ji L., Huang R. (2017). Social participation and perceived depression among elderly population in South Africa. Clin. Interv. Aging.

[78] Grundström J., Konttinen H., Berg N., Kiviruusu O. (2021). Associations between relationship status and mental well-being in different life phases from young to middle adulthood. SSM-Popul. Health.

[79] Bulloch A.G., Williams J.V., Lavorato D.H., Patten S.B. (2017). The depression and marital status relationship is modified by both age and gender. J. Affect. Disord..

[80] Rokach A., Matalon R., Rokach B., Safarov A. (2007). THE EFFECTS OF GENDER AND MARITAL STATUS ON LONELINESS OF THE AGED. Soc. Behav. Pers. Int. J..

[81] Thomeer M.B., Clark K.O. (2021). The development of gendered health-related support dynamics over the course of a marriage. J. Women Aging.

[82] Hossain B., Yadav P.K., Nagargoje V.P., Vinod Joseph K.J. (2021). Association between physical limitations and depressive symptoms among Indian elderly: Marital status as a moderator. BMC Psychiatry.

[83] Subathevan S., Suganthan S., Suranjith G.H.C., Dilshara H.M.K.S.J. (2022). Social and emotional loneliness among older adults in a coastal suburb in Sri Lanka. Aging Health Res..

[84] Pan L., Li L., Peng H., Fan L., Liao J., Wang M., Zhang Y. (2022). Association of depressive symptoms with marital status among the middle-aged and elderly in Rural China–Serial mediating effects of sleep time, pain and life satisfaction. J. Affect. Disord..

[85] Koenig H.G. (2012). Religion, Spirituality, and Health: The Research and Clinical Implications. ISRN Psychiatry.

[86] Hill T.D., Burdette A.M., Idler E.L., Settersten R.A., Angel J.L. (2011). Religious Involvement, Health Status, and Mortality Risk BT-Handbook of Sociology of Aging.

[87] Page R.L., Peltzer J.N., Burdette A.M., Hill T.D. (2020). Religiosity and Health: A Holistic Biopsychosocial Perspective. J. Holist. Nurs..

[88] Unterrainer H.-F., Ladenhauf K., Moazedi M., Wallner-Liebmann S., Fink A. (2010). Dimensions of Religious/Spiritual Well-Being and their relation to Personality and Psychological Well-Being. Pers. Individ. Differ..

[89] Rosmarin D.H., Leidl B., Rosmarin D.H. (2020). Chapter 3-Spirituality, Religion, and Anxiety Disorders. Koenig Religion, and Mental Health.

[90] Maraj H.A., Gülerce H., Rana S., Meraj M. (2020). Resilience and Hopelessness: Exploring the Mediator Role of Spirituality in the Global Situation of COVID-19. J. Kaji. Wil..

[91] Nery B.L.S., Cruz K.C.T., Faustino A.M., Santos C.T.B. (2018). Vulnerabilities, depression, and religiosity in the elderly hospitalised in an emergency unit. Rev. Gaúcha Enferm..

[92] de Jager Meezenbroek E., Garssen B., van den Berg M., van Dierendonck D., Visser A., Schaufeli W.B. (2012). Measuring Spirituality as a Universal Human Experience: A Review of Spirituality Questionnaires. J. Relig. Health.

[93] Fisher J. (2011). The Four Domains Model: Connecting Spirituality, Health and Well-Being. Religions.

[94] Burkhardt M.A. (1993). Characteristics of Spirituality in the Lives of Women in a Rural Appalachian Community. J. Transcult. Nurs..

[95] Chen Y.-H., Lin L.-C., Chuang L.-L., Chen M.-L., Msn Y.-H.C. (2017). The Relationship of Physiopsychosocial Factors and Spiritual Well-Being in Elderly Residents: Implications for Evidence-Based Practice. Worldviews Evidence-Based Nurs..

[96] Penny M.E., Meza K.S., Creed-Kanashiro H., Marin R.M., Donovan J. (2017). Fruits and vegetables are incorporated into home cuisine in different ways that are relevant to promoting increased consumption. Matern. Child Nutr..

[97] Joseph R.P., Ainsworth B.E., Mathis L., Hooker S., Keller C. (2017). Incorporating religion and spirituality into the design of community-based physical activity programs for African American women: A qualitative inquiry. BMC Res. Notes.

[98] Bożek A., Nowak P.F., Blukacz M. (2020). The Relationship Between Spirituality, Health-Related Behavior, and Psychological Well-Being. Front. Psychol..

[99] Arpino B., Gumà J., Julià A. (2018). Early-life conditions and health at older ages: The mediating role of educational attainment, family and employment trajectories. PLoS ONE.

[100] Ma J., Yang Y., Wan Y., Shen C., Qiu P. (2021). The influence of childhood adversities on mid to late cognitive function: From the perspective of life course. PLoS ONE.

[101] Daines C.L., Hansen D., Novilla M.L.B., Crandall A. (2021). Effects of positive and negative childhood experiences on adult family health. BMC Public Heath..

[102] Zhu H., Liao M., Carmona-Torres M., Cobo-Cuenca A.I., Laredo-Aguilera A., Ángel P. (2021). Childhood Circumstances and Mental Health in Old Age: A Life Course Survey in China. Public Health.

[103] Jia Z., Xu S., Zhang Z., Cheng Z., Han H., Xu H., Wang M., Zhang H., Zhou Y., Zhou Z. (2021). Association between mental health and community support in lockdown communities during the COVID-19 pandemic: Evidence from rural China. J. Rural Stud..

