# Supplementary material for: A Scoping Review on Biopsychosocial Predictors of Mental Health among Older Adults
Source: Int J Environ Res Public Health. 2022 Sep 1;19(17):10909. doi: 10.3390/ijerph191710909 (PMC9518331; doi:10.3390/ijerph191710909)
Supplement: Supplementary file 1 [file ijerph-19-10909-s001.zip › ijerph-1868443-supplementary.pdf]

International Journal of
Environmental Research and Public Health

MDPI

Supplementary Table S1. Summary of Characteristics of Identified Studies 1

No	Main author	Title	Year	Number of respondents	Study design	Location of study
1	Xu W.	Cognitive Impairment and Related Factors Among Middle-Aged and Elderly Patients with Type 2 Diabetes from a Bio-Psycho-Social Perspective	2021	240	Cross sectional	China
2	Bond L.	Biopsychosocial factors associated with depression and anxiety in older adults with intellectual disability: results of the wave 3 Intellectual Disability Supplement to The Irish Longitudinal Study on Ageing	2020	291	Cross sectional	Ireland
3	Olive Lisa S.	Fatigue, Physical Activity, and Mental Health in People Living with Inflammatory Bowel Disease, Fibromyalgia, and in Healthy Controls	2020	387	Cross sectional	Australia
4	Lindsay C.	Biopsychosocial predictors of perceived life expectancy in a national sample of older men and women	2017	6662	Cross sectional	England
5	Gang H.	Social participation and perceived depression among elderly population in South Africa	2017	442	Cross sectional	South Africa
6	Yi-Heng C.	The Relationship of Physio psychosocial Factors and Spiritual Well-Being in Elderly Residents: Implications for Evidence-Based Practice	2017	337	Cross sectional	Taiwan
7	Sara L.	Spirituality and quality of life in older adults: a path analysis model	2020	604	Cross sectional	Portugal
8	Blanco M.	Subjective well-being key elements of Successful Aging: A study with Lifelong Learners older adults from Costa Rica and Spain	2019	1293	Cross sectional	Costa Rica and Spain
9	Li Z.	Health lifestyles and Chinese oldest-old's subjective well-being—evidence from a latent class analysis	2021	3416	Cohort	China
10	Daniela V.	The role of spirituality and religiosity in subjective well-being of individuals with different religious status	2019	267	Cross sectional	Italy
11.	Xinlin C.	What intensity of exercise is most suitable for the elderly in China? A propensity score matching analysis	2021	9026	Cross sectional	China
12	Leonie S.	Mental health, smoking, harm reduction and quit attempts – a population survey in England	2020	5637	Cross sectional	England
13	Geum-Joon C.	Association Between Waist Circumference and Dementia in Older Persons: A Nationwide Population-Based Study	2019	872082	Cross sectional	South Korea

14	Xiaolei W.	Relationships among mental health, social capital and life satisfaction in rural senior older adults: a structural equation model	2022	245	Cross sectional	China
15	Christopher F. S.	Comparing a genetic and a psychological factor as correlates of anxiety, depression, and chronic stress in men with prostate cancer	2018	95	Cross sectional	Australia
16	Najada S.	Genetic Liability for Depression, Social Factors and Their Interaction Effect in Depressive Symptoms and Depression Over Time in Older Adults	2020	2279	Cross sectional	Netherlands
17	Alexandra W.	Perceived Social Support and Interpersonal Functioning as Predictors of Treatment Response Among Depressed Older Adults	2020	189	Cross sectional	United States of America
18	Ziyu J.	Association between mental health and community support in lockdown communities during the COVID-19 pandemic: Evidence from rural China	2021	3892	Cross sectional	China
19	Keqing Z.	Anxiety about aging, resilience and health Status among Chinese older adults: Findings from Honolulu and Wuhan	2020	824	Cross sectional	United States of America and China
20	Dzifa A.	Residence in urban and rural areas over the life course and depression among Ghanaian and South African older adults	2020	4199	Cross sectional	South Africa
21	Prince M. A.	Effect of cognitive and structural social capital on depression among older adults in Ghana: A multilevel cross-sectional analysis	2020	5573	Cross sectional	Ghana
22	Gialluisi A.	Lifestyle and biological factors influence the relationship between mental health and low-grade inflammation	2020	16952	Cohort	Italy
23	Joan D.	Loneliness and depression among older European adults: The role of perceived neighbourhood-built environment	2020	10799	Cross sectional	Finland, Poland, and Spain
24	Lob Eleonora	Adverse childhood experiences and depressive symptoms in later life: Longitudinal mediation effects of inflammation	2020	4382	Cohort	England
25	Naoko N.	A Pilot Study of the Relationship between Diet and Mental Health in Community Dwelling Japanese Women	2019	89	Cross sectional	Japan
26	Lara N.	Omega 3 Consumption and Anxiety Disorders: A Cross-Sectional Analysis of the Brazilian Longitudinal Study of Adult Health (ELSA-Brasil)	2018	12268	Cross sectional	Brazil
27	Jing M.	The influence of childhood adversities on mid to late cognitive function: From the perspective of life course	2021	9942	Cohort	China
28	Alexandra D.	Sleep quality components and mental health: Study with a non-clinical population	2018	1552	Cross sectional	Portugal, Spain, and Brazil

29	Jennifer C. D.	Examining the Inter-relations of Depression, Physical Function, and Cognition with Subjective Sleep Parameters among Stroke Survivors: A Cross-sectional Analysis	2019	72	Cross sectional	Canada
30	Aline R.	Loneliness is adversely associated with physical and mental health and lifestyle factors: Results from a Swiss national survey	2017	20007	Cross sectional	Swiss
31	Friederike H. B.	Loneliness as a gender-specific predictor of physical and mental health-related quality of life in older adults	2021	2171	Cohort	Germany
32	Maria Chaira F.	Are subjective cognitive complaints associated with executive functions and mental health of older adults?	2021	89	Case control	Italy
33	Nemenzo Endrex P.	Social Factors as Antecedents of Depression among Community-Dwelling Older Persons	2022	384	Cross sectional	Philippines
34	Fernández-Niñ Julian A.	Religious affiliation and major depressive episode in older adults: a cross-sectional study in six low- and middle- income countries	2019	21410	Cross sectional	China, Ghana, India, Mexico, Russia, and South Africa
35	Pravat B.	Lifestyle Behaviours and Mental Health Outcomes of Elderly: Modification of Socio-Economic and Physical Health Effects	2021	1280	Cross sectional	India
36	Hui Y.	Mental health and well-being in older women in China: implications from the Andersen model	2020	3527	Cross sectional	China
37	Huoyun Z.	Childhood Circumstances and Mental Health in Old Age: A Life Course Survey in China	2021	9750	Cohort	China
38	An T. M.	Factors Associated with Depression among the Elderly Living inUrban Vietnam	2018	299	Cross Sectional	Vietnam
39	Elody H.	Loneliness and Mental Health: The Mediating Effect of Perceived Social Support	2021	187	Cross Sectional	Netherlands
40	Thanh V.	Determining Risk for Depression among Older People Residing in Vietnamese Rural Settings	2019	523	Cross Sectional	Vietnam
41	Yanzhao C.	Latent class growth modelling of depression and anxiety in older adults: an 8-year follow-up of a population-based study	2021	3983	Cohort	Korea
42	Jeffrey L. B.	Depression and Multimorbidity: Considering Temporal Characteristics of the Associations Between Depression and Multiple Chronic Diseases	2019	2311	Cross Sectional	Canada
43	Jessica M. B.	Perceived Mindfulness and Depressive Symptoms Among People with Chronic Pain	2018	211	Cross Sectional	United States of America

44	Wendy J.	Gene–Environment Interplay Between Physical Exercise and Fitness and Depression Symptomatology	2020	756	Cross Sectional	Denmark
45	Abdul W.	Bipolar disorder among married women in Bangladesh: Survey in Rajshahi city	2020	279	Cross Sectional	Bangladesh
46	Wan-Qiu Y.	Gender differences in prevalence and clinical correlates of anxiety symptoms in first-episode and drug-naïve patients with major depressive disorder	2021	1718	Cross Sectional	China
47	Bruno A.	Early-life conditions and health at older ages: The mediating role of educational attainment, family and employment trajectories	2018	12034	Cross Sectional	Spain

2
